# Commuting by bicycle (vs. by car) is associated with improved aerobic power, microvascular function and diminished CO_2_ output in the atmosphere

**DOI:** 10.1113/EP092636

**Published:** 2025-08-29

**Authors:** Caterina Ursella, Giovanni Baldassarre, Lucrezia Zuccarelli, Federica Caponnetto, Francesco Curcio, Mattia D'Alleva, Maria De Martino, Elisabetta Fontanini, Andrea Palomba, Antonio Paolo Beltrami, Federico Formenti, Bruno Grassi

**Affiliations:** ^1^ Department of Medicine University of Udine Udine Italy; ^2^ Istituto di Patologia Clinica Azienda Sanitaria Universitaria Friuli Centrale (ASUFC) Udine Italy; ^3^ Nuffield Division of Anaesthetics University of Oxford Oxford UK; ^4^ Centre for Human and Applied Physiological Sciences, School of Basic and Medical Biosciences King's College London London UK; ^5^ Department of Biomechanics University of Nebraska Omaha Omaha Nebraska USA

**Keywords:** bicycle commuting, car commuting, CO_2_ output in atmosphere, endothelial function, human/environmental and exercise physiology, peak aerobic power, ${\dot{V}}_{O_{2}}$

## Abstract

The study investigated whether bicycle compared with car commuting, over relatively small distances, has positive effects on physiological variables, cardiometabolic fitness and CO_2_ output in the atmosphere. Bike Commuters (11 M, 15 F; age [median value (interquartile range)] 51.5 (38.3–56.8) years; body mass index [BMI] 22.8 (21.0–24.1) kg m^−2^) were compared with Car Commuters (12 M, 19 F; age 47.0 (36.0–56.5) years; BMI 23.5 (21.4–24.9) kg m^−2^). In a longitudinal arm of the study, 20 Car Commuters were re‐evaluated after they switched for 24 weeks to bicycle commuting (Car→Bike Commuters). Measurements included peak aerobic power (${\dot{V}}_{O_{2}\mathrm{peak}}$) and ventilatory thresholds on a cycle ergometer, blood flow increase in the common femoral artery during a passive leg movement (PLM) test, energy expenditure and ${\dot{V}}_{CO_{2}}$ exhaled during commuting. Bike Commuters had higher ${\dot{V}}_{O_{2}\mathrm{peak}}$ (33.7 (31.3–38.1) versus 25.3 (23.5–28.9) mL kg^−1^ min^−1^, *P *< 0.001) and ventilatory thresholds than Car Commuters, higher Δpeak blood flow (+25%, *P *= 0.04) and area under the blood flow versus time curve (+46%, *P *= 0.03) during PLM, and an enhanced skeletal muscle oxidative metabolism. ${\dot{V}}_{O_{2}\mathrm{peak}}$ and PLM variables increased in Car→Bike Commuters. Metabolic CO_2_ output during bicycle commuting was ∼12 times less than that for a petrol car. In moderately active individuals, short‐distance bicycle commuting at moderate intensity was associated, compared with car commuting, with positive effects on several physiological functions and environmental factors.

## Abstract (highlights)

- **What is the central question of this study?**
  What are healthy adults’ physiological responses to bicycle commuting, compared to car commuting, over ∼4.5 km and ∼17 min one‐way routes, 4–5 times per week? How much lower are CO_2_ emissions of bicycle compared to car commuting?
- **What insights does it reveal?**
  Bicycle commuting at a self‐selected moderate speed was associated with a more favourable body composition, greater aerobic power and exercise tolerance, and improved microvascular/endothelial function and skeletal muscle oxidative metabolism. CO_2_ output in the atmosphere was 12 times lower with bicycle commuting.

## 1 INTRODUCTION

Several studies have investigated the influence of the urban built environment on human habits and behaviour, the consequences for individuals’ physical activity quantity and quality, and the impact on personal and public health (Ewing & Cervero, 2010; Frank & Engelke, 2001; Handy et al., 2002). Life in modern cities is often characterized by a sedentary lifestyle and physical inactivity. It is widely acknowledged that physical inactivity is associated with an increased risk of chronic health conditions such as diabetes, cardiovascular diseases, obesity, cancer and several other diseases (Booth et al., 2017; Lee et al., 2012; Pinhas‐Hamiel & Zeitler, 2005; Tamayo et al., 2014). The pathophysiological mechanisms linking inactivity and chronic disease development include, among others, reduced skeletal muscle mass and impaired function (sarcopenia), insulin resistance, endothelial/microvascular and mitochondrial dysfunction, and increased oxidative stress and damage (Booth et al., 2017). On the other hand, the role of exercise in the prevention and treatment of chronic diseases is well established (Heath et al., 2012; Pendersen & Saltin, 2006). In a recent epidemiological study (Friel et al., 2024), walking or bicycle commuting was demonstrated to exert positive effects on life expectancy and in the prevention of cardiovascular, neoplastic, mental health conditions and other diseases. Even very small exercise ‘doses’ may have a beneficial effect. In a recent study, for example (Duran et al., 2023), breaking up prolonged sitting by a few minutes of light‐intensity walking every hour was enough to improve glycaemic and blood pressure control.

Besides favouring a sedentary behaviour, car usage for private transportation is a source of CO_2_ and other pollutant emissions in the atmosphere, with profoundly negative consequences on human health, climate change and the environment (Nieuwenhuijsen & Khreis, 2016). In the 1990s urban planning policies started adopting strategies in which the hierarchy between cars and pedestrians was inverted, in order to promote active mobility (walking, cycling) and discourage car use in urban areas with traffic distances under 5 km (Dorato, 2020). The urban model of the ‘15‐min city’ promotes active mobility transportation within high density urban environments: in this model individuals can reach primary services and workplaces by cycling within a time limit of 15 min, covering about 5 km (Graells‐Garrido et al., 2021).

The beneficial effects of active commuting on individuals’ health have been investigated (Friel et al., 2024; Saunders et al., 2013). Interventional studies on the physiological effects of cycling demonstrated that a one‐way trip commuting distance of 7–15 km, requiring moderate‐intensity aerobic activity, was associated with improved cardiorespiratory and metabolic fitness, improved physical performance, and positive effects on coronary heart disease risk factors (De Geus et al., 2007, 2008; Hendriksen et al., 2000; Oja et al., 1991). It has been demonstrated that cycling to work or to school, covering distances such as those mentioned above at a self‐selected intensity, meets the American College of Sports Medicine's minimal recommendations for a healthy lifestyle (De Geus et al., 2007). Other studies investigated bicycle commuting and the associated improvement in health and quality of life from a self‐reported point of view (De Geus et al., 2008; Neumeier et al., 2020).

In the present study we compared bicycle commuting versus car commuting over a 4–5 km one‐way distance (shorter than the distances of the studies mentioned above), performed at moderate intensity 4–5 times per week, in the environment of a typical northern Italian mid‐size town (Udine). The exercise training stimulus was chosen to be of moderate intensity. The geographical area is characterized by a low demographic density of 1722 inhabitants km^−2^ (ISTAT, 2024) and should represent an ideal case study of a car‐dependent territory (Cucca & Tacchi, 2012; Saeidizand et al., 2022).

The specific aim of the study was to investigate, in bike commuters versus car commuters, physiological variables associated with the risk of development of cardiovascular, metabolic and other diseases. As indices of cardiorespiratory fitness and exercise tolerance we investigated variables such as the peak aerobic power (peak O_2_ uptake, ${\dot{V}}_{O_{2}\mathrm{peak}}$) (Myers et al., 2002), and the ventilatory thresholds (Wasserman & Whipp, 1975). In the search for pathophysiological factors potentially responsible for the impairments we non‐invasively investigated microvascular/endothelial function (Gifford & Richardson, 2017) and skeletal muscle oxidative metabolism (Grassi & Quaresima, 2016). Blood‐derived variables and circulating metabolites and biomarkers associated with inflammation and cardiovascular disease, as well as body composition and the presence/absence of sarcopenia, were also investigated. Another specific aim of the study was to quantify the reduction of CO_2_ emission in the atmosphere occurring during bike commuting versus car commuting. Such a comprehensive evaluation, covering both physiological and environmental aspects, is lacking in the literature, particularly for short commutes.

## 2 METHODS

### 2.1 Ethics

The study was approved by the Institutional Review Board of the Department of Medicine at the University of Udine (reference number 145/2022). All methods were performed in accordance with the standards outlined in the *Declaration of Helsinki*. Informed written consent was obtained from all participants before taking part in the experiments. The participants did not receive any remuneration.

### 2.2 Participants

**FIGURE 1:**
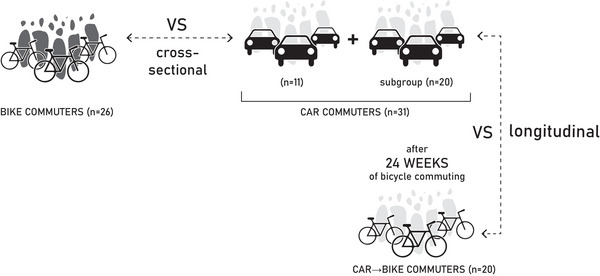
Structure of study design. Participants of group Bike Commuters are represented by dark grey silhouettes with bicycle icons. Participants of group Car Commuters are represented by light grey silhouettes with car icons. In the cross‐sectional study, group Bike Commuters (*n* = 26) was compared with group Car Commuters (*n* = 31). In the longitudinal study a subgroup of Car Commuters (*n* = 20) was evaluated before and after (Car→Bike Commuters) 24 weeks of bicycle commuting.

A cross‐sectional comparison was performed between a group of healthy participants habitually commuting by bicycle (Bike Commuters) and a group of healthy participants habitually commuting by car (Car Commuters) (Figure 1). A longitudinal approach was also followed, by re‐examining a subgroup of Car Commuters after 24 weeks during which they agreed to commute by bicycle Car→Bike Commuters (Figure 1).

For the Bike Commuters group, we recruited 26 participants (11 males and 15 females, age [median values (interquartile range)] 51.5 (38.3–56.8) years, body mass index [BMI] 22.8 (21.0–24.1) kg m^−2^), who regularly commuted by bicycle for at least 1 year before entering the study. For the Car Commuters group, we recruited 31 participants (12 males and 19 females, 47.0 (36.0–56.5) years, BMI 23.5 (21.4–24.9) kg m^−2^), who regularly commuted by car. For the longitudinal arm of the study, 20 participants of the Car Commuters group (seven males and 13 females, 50.5 (35.8–57.0) years, BMI 23.4 (21.1–25.1) kg m^−2^) agreed to change commuting habits from car to bicycle for 24 weeks (Car→Bike Commuters), and were re‐evaluated after the 24‐week period. The recruitment was conducted with a survey that was disseminated (by the local press and online) with the collaboration of the Communication Office of the Department of Medicine of the University of Udine, of the office ‘Uniud sostenibile’ of the University, and the local cycling club FIAB Udine—aBcitUdine. The survey investigated commuting habits, lifestyle and levels of physical activity of the participants. Eligible participants were selected according to the inclusion/exclusion criteria mentioned below.

Inclusion criteria were the following: BMI between 18.5 kg m^−2^ and 30 kg m^−2^; age between 19 and 65 years; physical activity levels, apart from bicycle commuting, not exceeding ACSM recommendations of 150 min week^−1^ of moderate‐ and 75 min week^−1^ of vigorous‐intensity cardiorespiratory exercise (Garber et al., 2011), and normal blood pressure. Physically very active subjects, subjects undergoing training protocols and athletes were excluded. The participants had to work and live in Udine or in the surrounding municipalities and had to commute from home to work and back at least three times a week, for distances ≥2 km one‐way. Exclusion criteria also included smoking and the presence of significant neurological/cerebrovascular, cardiovascular, pulmonary, rheumatological, metabolic or renal pathologies.

Participants’ habitual physical activity levels were assessed at the beginning of the study by the self‐reported International Physical Activity Questionnaire Short Form IPAQ‐SF (Lee et al., 2011). The specific types of activity that were included are walking, moderate‐intensity activities and vigorous intensity activities, undertaken across four domains (i.e., leisure time, domestic and gardening activities, work‐related, and transport‐related activities). The method weights each type of activity by its energy requirements, defined in METs (multiples of the resting metabolic rate), yielding an overall score evaluating the habitual level of physical activity, expressed in MET‐min week^−1^. For Bike Commuters, two assessments were performed: one including bicycle commuting and one excluding bicycle commuting.

Bike Commuters had been regularly commuting by bicycle for at least a year before the beginning of the study, for at least three times a week and with a non‐pedal‐assisted or electric bicycle. Car Commuters were regularly commuting by car at the beginning of the study and owned a non‐pedal‐assisted or a non‐electric bicycle to be used in the longitudinal study. Participants of the Car Commuters group agreed to share the GPS tracks of their home–work activities during the 24 weeks of the longitudinal study intervention, which were recorded using the Polar Flow App and a personal smartphone. Data were checked weekly by the researchers. In order to confirm the reported activity, participants of the Bike Commuters and Car→Bike Commuters groups monitored the intensity of their home–work commuting activity by bicycle, during the first week of intervention, by a chest strap (Polar H7, Polar Electro Oy, Kempele, Finland). Considered variables were heart rate (HR), speed, time and distance.

### 2.3 Measurements

Experiments were performed in the Exercise Physiology Laboratory, Department of Medicine, University of Udine. All tests were performed under continuous medical supervision and following standard safety procedures. Before the tests the participants underwent a standard physical examination including a medical history.

#### 2.3.1 Health related quality of life survey SF‐36 questionnaire

The evaluation of the self‐stated health status and quality of life was performed by the Health‐Related Quality of Life (HRQoL) SF‐36 questionnaire (Ware, 2000). The questionnaire is a short‐form health survey composed by 36 questions, which aim to describe the individual health status in eight domains, half of which are related to the Physical Health Summary Measure, whereas the remaining half are related to the Mental Health Summary Measure. The score for each domain is expressed in a scale from 0 to 100%, with higher scores indicating a better health status (Ware et al., 1998).

#### 2.3.2 Anthropometry and body composition

Body mass, height and BMI were determined. The percentages of fat mass (%FM) and fat‐free mass (%FFM) were determined by whole body bioimpedance analysis (BIA). BIA was performed by a phase sensitive single frequency device (BIA 101 BIVA, Akern srl, Florence, Italy), following standard procedures (Lukaski, 1987). Leg circumferences and heights, together with skinfold measurements by a calliper (Gima, Milan, Italy), were collected to estimate thigh volume (Jones & Pearson, 1969). Quadriceps femoris muscle mass was then calculated by utilizing the following equation (Andersen & Saltin, 1985): Quadriceps femoris muscle mass (kg) = 0.307 × thigh volume (L) + 0.353.

#### 2.3.3 Evaluation of endothelial/microvascular functions

Blood flow in the common femoral artery was estimated by measurements of blood flow velocity and vessel diameter distally to the inguinal ligament, 2.0–2.5 cm proximally to the bifurcation of the superficial and deep femoral artery, by using an ultrasound system (Versana Active, GE Healthcare, Milwaukee, WI, USA) with a linear array transducer operating at the imaging frequency of 9 MHz. Two‐dimensional measurements of the arterial lumen were made from B‐mode images in a longitudinal view. Measurements of the vessel diameter were taken at the same time point in the cardiac cycle (peak of the R wave, derived from the integrated ECG system). Blood flow velocities were collected with the sample volume covering more than 75% of the arterial lumen, and with the insonation angle always kept ≤60°. Arterial blood flow was automatically calculated second by second by the software available in the ultrasound system, by multiplying arterial cross‐sectional area and mean blood flow velocity.

Blood flow measurements were performed at rest and during 1 min of PLM of the right leg, following provided guidelines (Gifford & Richardson, 2017). The subjects remained seated with their legs extended and supported for 15 min before data collection. Resting echo‐Doppler data were recorded for about 2 min and were followed by measurements performed during 60 s of cyclical passive knee extension and flexion movements. The movements were performed across a 90° range of motion (180°–90°–180°) at 1 Hz, following a metronome. The same trained researcher manually moved the subject's leg in all groups of participants. The subjects were instructed not to activate their muscles during the movements. The absence of active movements was ensured during preliminary practice runs. The same researcher performed the echo‐Doppler measurements in all groups of subjects.

Measurements were performed in duplicate, and the protocol was repeated after 15 min of recovery. Measurements obtained during the two repetitions were superimposed and average values were obtained for each subject and retained for data analysis. Resting blood flow and peak blood flow during PLM were calculated. The area under the blood flow versus time response (area under the curve, AUC) was obtained by calculating the integral of the function over the entire 60 s, after subtracting the resting baseline value (see Gifford & Richardson, 2017).

#### 2.3.4 Incremental cycle ergometer test

Participants performed an incremental test on a cycle ergometer (Monark 818E; Stockholm, Sweden) in the late morning, a few hours after a standard breakfast. They were asked to keep a constant cadence between 60 and 70 revolutions per minute throughout the test. The protocol consisted of a 3‐min low‐intensity constant load exercise, followed by an incremental exercise (increasing the work rate every minute by 7, 10, 12 or 15 W increases, according to the participants’ estimated fitness level) conducted until voluntary exhaustion (inability to maintain the required pedalling rate), which was reached in 8–12 min (Wasserman & Whipp, 1975).

During the test, respiratory and gas exchange parameters (i.e. pulmonary ventilation [${\dot{V}}_{E}$], tidal volume [*V*_T_], breathing frequency [*f*_R_], ${\dot{V}}_{O_{2}}$, and carbon dioxide output [${\dot{V}}_{CO_{2}}$]) were assessed breath‐by‐breath by a metabolic cart (Quark PFTergo, Cosmed, Rome, Italy). Expiratory flow measurements were performed by a turbine flowmeter, calibrated before each experiment by a 3‐L syringe at three different flow rates. Calibration of O_2_ and CO_2_ analysers was performed before each experiment by utilizing gas mixtures of known composition. The respiratory exchange ratio (RER) was calculated as ${\dot{V}}_{CO_{2}}$/${\dot{V}}_{O_{2}}$. Heart rate (HR) was recorded using a 12‐lead electrocardiograph (Quark C12x, Cosmed, Rome, Italy). At the end of exercise, the rate of perceived exertion (RPE) was determined using the Borg 6–20 scale (Borg, 1970).

Oxygenation changes of vastus lateralis of the right leg were determined by a portable continuous‐wave spatially resolved near infrared spectroscopy (NIRS) instrument (PortaMon; Artinis Medical System, Elst, The Netherlands) (Grassi & Quaresima, 2016). The instrument non‐invasively measures micromolar changes in oxygenated haemoglobin (Hb) + myoglobin (Mb) concentrations (∆[oxy(Hb+Mb)]) and in deoxygenated [Hb+Mb] (∆[deoxy(Hb+Mb)]), with respect to an initial value arbitrarily set equal to zero, obtained during the resting condition preceding the test. The sampling frequency was set at 10 Hz. An increased ∆deoxy(Hb+Mb)] or a decreased ∆[oxy(Hb+Mb)] indicate an increased fractional O_2_ extraction in the tissue under consideration (Grassi & Quaresima, 2016). The ∆[deoxy(Hb+Mb)] signal was considered in the present study since it is usually less affected (with respect to the ∆[oxy(Hb+Mb)] signal) by changes in blood volume in the tissue (Grassi & Quaresima, 2016). The probe was firmly attached to the skin overlying the lower third of the quadriceps femoris muscle of the right thigh. Adipose tissue thickness (ATT) at the site of NIRS probe application was measured by a caliper (Gima, Milan, Italy). Skinfold thickness ≤20 mm was considered as the cut‐off for NIRS utilization. Black bandages were put around the probe and the skin to prevent contamination from ambient light. ∆deoxy(Hb+Mb)] data were expressed as a percentage of the maximal muscle deoxygenation obtained at rest by inflating a pressure cuff (∼300 mmHg) positioned at the inguinal crease of the thigh. This manoeuvre typically lasts a few minutes, until ∆deoxy(Hb+Mb)] reaches a plateau, which represents an index of the maximal fractional O_2_ extraction by skeletal muscle (Grassi & Quaresima, 2016).

Mean values of cardio‐respiratory and metabolic responses to exercise were calculated during the last 20 s of each minute of exercise, and values obtained during the exhausting work rate were considered peak values. ${\dot{V}}_{O_{2}\mathrm{peak}}$, that is the aerobic power reached at peak work rate, was considered an overall index evaluating the maximal performance of the cardiorespiratory system. The gas exchange threshold (GET) was determined using both the ‘V‐slope’ method and secondary criteria (Beaver et et al., 1986); the respiratory compensation point (RCP) was identified by standard criteria (Wasserman & Whipp, 1975).

#### 2.3.5 Measurements in the field: Energy expenditure and CO_2_ output

${\dot{V}}_{E}$, ${\dot{V}}_{O_{2}}$ and ${\dot{V}}_{CO_{2}}$ were determined ‘breath‐by‐breath’ during typical commutes by bicycle or by car by a portable metabolimeter (K5; Cosmed). Expiratory flow measurements were performed by a turbine flowmeter, calibrated before each experiment by a 3‐L syringe at three different flow rates. Calibration of O_2_ and CO_2_ analysers was performed before each experiment by utilizing gas mixtures of known composition.

All participants ate a standard breakfast before the morning commute. The afternoon commute was performed a few hours after a standard lunch. HR was simultaneously determined by a chest strap (Polar H10, Polar Electro Oy). Global positioning system (GPS) tracking was determined by the portable metabolimeter integrated GPS sensor (10 Hz frequency, 2.5 m position accuracy, 0.1 m s^−1^ speed accuracy). These data allowed us to calculate the distance covered and the instantaneous and mean speed of locomotion of the participants.

The energy expenditure (expressed in kcal) associated with the commute was estimated by indirect calorimetry from the total mean ${\dot{V}}_{O_{2}}$ (L min^−1^), multiplied by the duration of the commuting, and then multiplied by 5, after considering an energy equivalence of 5 kcal per litre of consumed O_2_. This conversion is utilized in participants undergoing light to moderate steady‐state exercise (Scott, 2005).

Metabolic CO_2_ emission of the participants in the atmosphere during the commutes was estimated based on the ${\dot{V}}_{CO_{2}}$ determined by the portable metabolimeter. The total 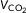 (in litres) exhaled during the trip was calculated by multiplying ${\dot{V}}_{CO_{2}}$ by the duration of the trip. 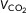 was then divided by the unit of distance (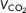 km^−1^). 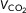 per unit of distance was then expressed in g km^−1^ after considering that 1 mol of CO_2_ (=44 g) occupies 24.056 L at standard barometric pressure and at an ambient temperature of 20°C (Minetti & Pavei, 2011).

At the end of the test, the RPE was determined using the Borg 6–20 scale (Borg, 1970).

#### 2.3.6 Venous blood samples

During the same week in which the measurements in the laboratory were performed, venous samples were collected. In the days prior to venipuncture, patients were instructed on how to prepare for venous blood collection. Blood samples were collected in dedicated ambulatories of the University Hospital of Udine, following the written procedures in force at the institution, which are based on the latest guidelines of the European Federation of Clinical Chemistry and Laboratory Medicine (EFLM) and the Clinical and Laboratory Standards Institute (CLSI). Blood samples were taken in the early morning before breakfast. All determinations were performed employing the same routine methodologies, and the same standards in use in the clinical laboratory of the institution. The only test which was employed that is not used in routine was the syndecan‐1 ELISA (Novus Biologicals, Centennial, CO, USA). In this case, the analysis was conducted on plasma samples following the manufacturer's instructions. Parameters associated with cardiovascular risk (total cholesterol, low‐density lipoprotein [LDL]‐cholesterol, high‐density lipoprotein [HDL]‐cholesterol, triglycerides, lipoprotein(a), homocysteine, troponin I), glycaemic control (haemoglobin A1c, DCCT), coagulation state (fibrinogen), inflammation (interleukin‐6), endothelial function (pro‐adrenomedullin) and glycocalyx status (syndecan‐1) were determined.

### 2.4 Statistical analysis

Since most of the data did not present a normal distribution by the Shapiro–Wilk test, non‐parametric statistical tests were utilized for all comparisons. Data are presented as median values and interquartile range. The statistical significance of differences was checked by the Mann–Whitney test in the cross‐sectional study (Bike Commuters vs. Car Commuters) and by the Wilcoxon test in the longitudinal study (Car→Bike Commuters vs. Car Commuters). The level of statistical significance was set at 0.05. A power analysis was performed a priori in order to estimate the number of participants to be included in the study. Taking ${\dot{V}}_{O_{2}\mathrm{peak}}$ as the primary endpoint, and literature data (Wilmore et al., 1980) for sedentary and active individuals, a statistical power of 80% with an alpha error of 0.05, the needed sample size was 25 participants per group. Statistical analyses were carried out by a commercially available software package (Prism 8.0; GraphPad Software, San Diego, CA, USA).

## 3 RESULTS

### 3.1 Habitual level of physical activity

Home–work distance was not different between Bike Commuters and Car Commuters (3.9 (2.8–2.8) versus 4.6 (2.8–6.7) km one‐way trip; *P *= 0.25). The habitual physical activity score, evaluated by the IPAQ‐SF questionnaire, was higher in Bike Commuters versus Car Commuters (1042 (738–1244) MET min week^−1^ versus 552 (338–690) *P *< 0.001). According to the IPAQ classification (Lee et al., 2011), 21 participants of the Bike Commuters group were in the ‘minimally active’ category, and five in the ‘inactive’ category. In the Car Commuters group 15 participants were in the ‘minimally active’ and 16 participants were in the ‘inactive’ category. The difference in habitual physical activity between Bike Commuters and Car Commuters disappeared when the physical activity associated with bicycle commuting was not taken in consideration. In this case, indeed, the scores were 678 (407–968) MET min week^−1^ in Bike Commuters versus 562 (328–654) in Car Commuters (*P *= 0.19).

### 3.2 Health related quality of life (HRQoL) survey: SF‐36 questionnaire

The results of the questionnaire are given in Table 1 (higher scores indicate a better status). For the Physical Functioning variable, the score was lower in Car Commuters versus Bike Commuters (*P *= 0.01). Also for the Mental Health variable the score was lower in Car Commuters versus Bike Commuters (*P *= 0.01); this score increased in Car→Bike Commuters (*P *= 0.01). For both the Physical Component domains and the Mental Component domains of the questionnaire, the Summary Evaluation (last two lines of the Table) showed no differences in the cross‐sectional and in the longitudinal comparisons (*P *= 0.67, 0.87, 0.14 and 0.12, respectively).

**TABLE 1:** Health related quality of life (HRQoL) survey: SF‐36 questionnaire.

	Bike commuters (*n* = 26)	Car commuters (*n* = 31)	*P*	Car commuters (*n* = 20)	Car→Bike commuters (*n* = 20)	*P*
Males	11	12		7		
Females	15	19		13		
Physical functioning	100.0 (100.0–80.0)	95.0 (90.0–97.5)	0.01	95.0 (90.0–100.0)	100.0 (95.0–100.0)	0.08
Role limitations due to physical health problems	100.0 (100.0–0)	100.0 (100.0–100.0)	0.97	100.0 (100.0–100.0)	100.0 (93.8–100.0)	0.60
Bodily pain	90.0 (77.5–45.0)	90.0 (77.5–100.0)	0.48	90.0 (76.9–90.0)	90.0 (87.5–100.0)	0.01
General health perception	65.0 (60.0–45.0)	65.0 (60.0–75.0)	0.48	67.5 (58.8–76.3)	72.5 (60.0–81.3)	0.14
Vitality	63.8 (58.8–30.0)	58.8 (50.0–66.3)	0.17	59.4 (49.7–65.0)	62.5 (53.8–75.0)	0.19
Social functioning	87.5 (75.0–37.5)	75.0 (62.5–87.5)	0.47	75.0 (62.5–100.0)	87.5 (71.9–100.0)	0.62
Role limitations due to emotional problems	100.0 (66.7–0)	100.0 (50.0–100.0)	0.33	83.3 (58.3–100.0)	100.0 (66.7–100.0)	0.57
Mental health	76.0 (68.0–45.6)	64.0 (54.8–78.0)	0.01	62.8 (49.6–69.0)	76.0 (63.0–84.0)	0.01
Physical component summary	54.8 (52.8–41.6)	54.9 (51.2–57.4)	0.74	55.8 (52.1–57.9)	56.0 (53.2–58.3)	0.87
Mental component summary	50.6 (44.7–29.2)	46.2 (35.1–53.1)	0.14	45.2 (33.2–49.9)	50.6 (41.7–53.9)	0.12

Health related quality of life survey: SF‐36 questionnaire. Median and interquartile range values for variables related to the ‘physical functioning’ and ‘mental component’ domains, obtained in the different groups of participants. For both the ‘Physical Component’ domains and the ‘Mental Component’ domains the ‘Summary evaluations’ (last two lines of the Table) showed no significant differences in the cross‐sectional and in the longitudinal comparisons. See text for further details.

### 3.3 Anthropometry and body composition

No differences were observed between Bike Commuters and Car Commuters, or between Car→Bike Commuters and Car Commuters, in terms of body mass (*P *= 0.93 and 0.53, respectively), height (*P *= 0.27) and BMI (*P *= 0.26 and 0.57, respectively) (see Table 2). In the cross‐sectional analysis, the %FM was higher (*P *= 0.01) and the %FFM was lower (*P *= 0.01) in Car Commuters versus Bike Commuters. No differences for these variables were observed between Car→Bike Commuters and Car Commuters (*P *= 0.19 and 0.21, respectively).

**TABLE 2:** Anthropometry and body composition.

	Bike commuters (*n* = 26)	Car commuters (*n* = 31)	*P*	Car commuters (*n* = 20)	Car→Bike commuters (*n* = 20)	*P*
Males	11	12		7	—	
Females	15	19		13	—	
Age (years)	51.5 (38.3–56.8)	47.0 (36–56.5)	0.45	50.5 (35.8–57.0)	—	—
Body mass (kg)	65.5 (60–75.5)	65.5 (60–75.5)	0.93	63.5 (58.3–73.8)	63.5 (56.8–71.6)	0.53
Height (m)	1.71 (1.65–1.78)	1.7 (1.61–1.79)	0.27	1.65 (1.61–1.72)	—	
BMI (kg m^−2^)	22.8 (21.0–24.1)	23.5 (21.4–24.9)	0.26	23.4 (21.1–25.1)	23.6 (21.3–24.9)	0.57
%FM	21.5 (17.2–26.2)	26.1 (21.3–31.6)	0.01	24.6 (19.9–30.7)	26.0 (20.4–32.2)	0.19
%FFM	78.5 (73.8–82.8)	73.9 (68.4–78.8)	0.01	75.5 (69.3–80.2)	74.1 (67.9–79.7)	0.21
Waist circumference (cm)	78.3 (74.6–84.5)	78.0 (74–87.9)	0.30	77.5 (73.8–87.2)	81.0 (74.0–84.0)	0.51
Thigh volume (mL)	5109 (4168–5843)	4232 (3418–5351)	0.07	4109 (3313–5380)	4637 (3578–5324)	0.15
Thigh mass (kg)	1.9 (1.6–2.15)	1.7 (1.4–2**.0**)	0.07	1.6 (1.4–2.0)	1.8 (1.5–2.0)	0.15

Median and interquartile range values for the anthropometric variables obtained in the different groups of participants. See text for further details. BMI, body mass index; %FM, percentage of fat mass;%FFM, percentage of fat‐free mass.

### 3.4 Evaluation of endothelial/microvascular functions

**FIGURE 2:**
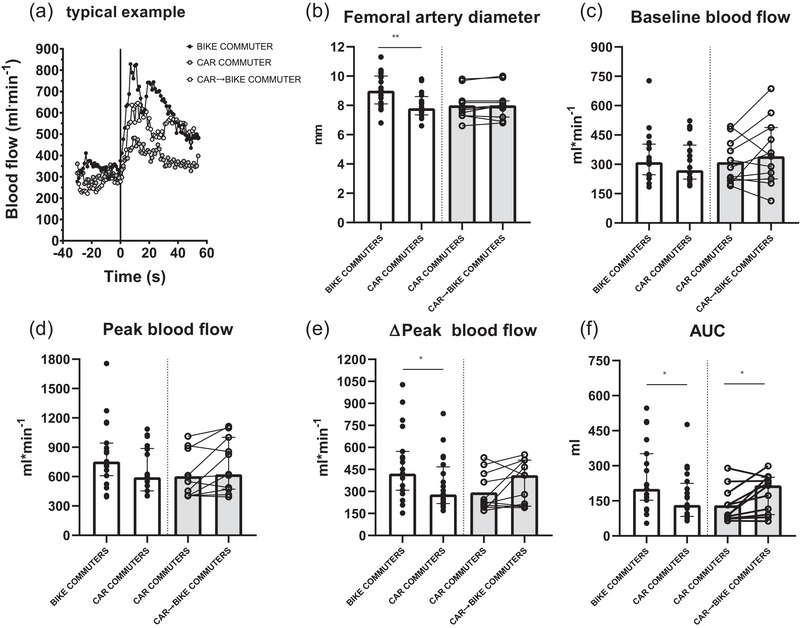
Hyperaemic response during passive leg movement: evaluation of microvascular/endothelial function. (a) A typical example of blood flow vs. time tracings, where the vertical line indicates the onset of passive leg movement. (b–e) Individual and median and interquartile range data are presented for femoral artery diameter (b), baseline blood flow (c), peak blood flow (d), Δpeak blood flow (e), and area under the blood flow versus time course (AUC) (f), in the different groups of participants. For the cross‐sectional comparison: Bike Commuters, *n* = 26 versus Car Commuters, *n* = 31. For the longitudinal comparison: Car Commuters *n* = 20 versus Car→Bike Commuters, *n* = 20. See text for further details. Asterisks denote differences between groups by means of Student's unpaired *t*‐test: *P < 0.05, **P < 0.01.

Typical examples of the blood flow versus time tracing during the PLM test are shown for the three groups of participants in Figure 2a. The figure presents also median and interquartile range values obtained in the groups for the different variables. The diameter of the common femoral artery was significantly greater in Bike Commuters than in Car Commuters (*P *< 0.01); no difference was observed between Car→Bike Commuters and Car Commuters (*P *= 0.002). Whereas baseline blood flow values were not different in the various groups (*P *= 0.55 and 0.32, respectively), the hyperaemic response during PLM (peak blood flow, Δpeak blood flow [*P *< 0.05], AUC [*P *< 0.05]) was more pronounced in Bike Commuters versus Car Commuters. For peak blood flow the difference did not reach statistical significance (*P *= 0.12 in cross‐sectional comparison and *P *= 0.053 for longitudinal comparison). For the longitudinal comparison AUC was higher in Car→Bike Commuters versus Car Commuters (*P *< 0.05). No differences were observed for other variables.

### 3.5 Incremental cycle ergometer test

**FIGURE 3:**
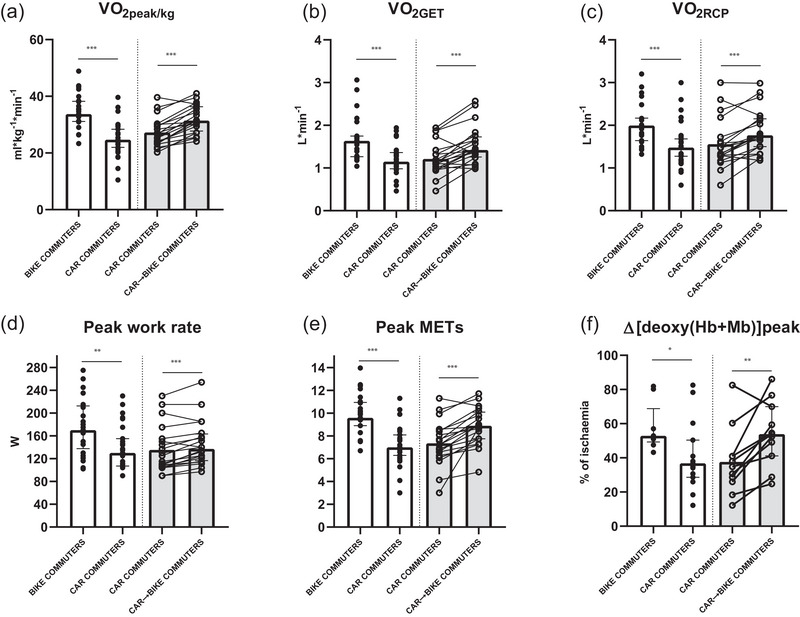
Cardiorespiratory fitness, exercise tolerance and skeletal muscle oxidative metabolism during the incremental exercise test on a cycle ergometer. Individual and median and interquartile range data are presented for the different groups of participants. For the cross‐sectional comparison: Bike Commuters, *n* = 26 versus Car Commuters, *n* = 31. For the longitudinal comparison: Car Commuters, *n* = 20 versus Car→Bike Commuters *n* = 20. (a, d–f) Peak values for oxygen uptake ${\dot{V}}_{O_{2}\mathrm{peak}}$ (a), work rate (d), METs (e), and fractional O_2_ extraction by skeletal muscle Δ[deoxy(Hb+Mb)] (f). (b, c) Oxygen uptake at the gas exchange threshold ${\dot{V}}_{O_{2}\mathrm{GET}}$ (b) and at the respiratory compensation point ${\dot{V}}_{O_{2}\mathrm{RCP}}$ (c). See text for further details. Asterisks denote differences between groups by means of Student's unpaired *t*‐test: *P < 0.05, **P < 0.01, ***P < 0.001.

Variables determined at peak exercise during the incremental test on the cycle ergometer are presented in Table 3. Some relevant variables are also presented in Figure 3. Peak work rate and ${\dot{V}}_{O_{2}\mathrm{peak}}$ (expressed in L min^−1^ and in mL kg^−1 ^min^−1^) were lower in Car Commuters versus Bike Commuters (*P *< 0.001, <0.001 and <0.001, respectively). ${\dot{V}}_{O_{2}\mathrm{peak}}$ increased in Car→Bike Commuters versus Car Commuters (*P *< 0.001). The same pattern was observed for the ventilatory thresholds (GET and RCP). In all conditions, ${\dot{V}}_{O_{2}}$ at GET and ${\dot{V}}_{O_{2}}$ at RCP were ∼70% and ∼85%, respectively, of ${\dot{V}}_{O_{2}\mathrm{peak}}$. METs number at peak exercise was 9.6 in Bike Commuters and 7.0 in Car Commuters (*P *< 0.001); METs at peak exercise increased to 8.9 in Car→Bike Commuters (*P *< 0.001). Δ[deoxy(Hb+Mb)]_peak_ was lower in Car Commuters versus Bike Commuters (*P *= 0.01) and increased in Car→Bike Commuters versus Car Commuters (*P *= 0.005). Skin fold thickness at the site of placement of the NIRS probe ranged between 7.1 and 15.6 mm; mean values were not different between Bike Commuters and Car Commuters (*P *= 0.37), or between Car→Bike Commuters and Car Commuters (*P *= 0.53).

**TABLE 3:** Incremental cycle ergometer test.

	Bike commuters (*n* = 26)	Car commuters (*n* = 31)	*P*	Car commuters (*n* = 20)	Car→Bike commuters (*n* = 20)	*P*
Values at peak						
Work rate (W)	170 (145–210)	130 (108–153)	0.009	120 (107–152)	137 (120–160)	0.001
${\dot{V}}_{O_{2}}$ (L min^−1^)	2.326 (2.016–2.666)	1.723 (1.432–2.063)	<0.001	1.647 (1.428–2.021)	1.927 (1.676–2.347)	<0.001
${\dot{V}}_{O_{2}}$ (mL kg min^−1^)	33.7 (31.2–38.1)	25.3 (23.5–28.8)	<0.001	26.0 (23.6–29.8)	31.4 (28.8–35.9)	<0.001
${\dot{V}}_{CO_{2}}$ (L min^−1^)	2.540 (2.156–2.975)	1.982 (1.563–2.386)	0.015	1.805 (1.565–2.263)	2.199 (1.938–2.642)	<0.001
RER	1.08 (1.06–1.12)	1.12 (1.07–1.15)	0.045	1.12 (1.07–1.16)	1.14 (1.09–1.15)	0.84
${\dot{V}}_{E}$ (L min^−1^)	84.2 (58.1–95.8)	58.5 (47.6–74.6)	0.011	55.7 (48.4–69.8)	73.3 (61.4–83.1)	<0.001
*V*_T_ (L)	2.424 (1.911–2.722)	1.804 (1.563–2.393)	0.058	1.797 (1.572–2.493)	2.064 (1.777–2.607)	<0.001
*f*_R_ (breaths min^−1^)	32.52 (31.23–36.29)	30.38 (25.31–35.39)	0.120	29.26 (25.55–34.14)	34.79 (28.01–40.39)	0.002
$P_{\mathrm{ET}O_{2}}$ (mmHg)	108.8 (106.4–111.5)	110.6 (106.3–112.1)	0.764	110.6 (104.7–112.0)	112.2 (108.5–115.1)	0.014
$P_{\mathrm{ETC}O_{2}}$ (mmHg)	39.1 (37.3–42.4)	38.8 (36.9–43.5)	0.932	39.1 (37.1–44.1)	37.9 (34.9–40.8)	0.003
HR (b min^−1^)	169 (156–177)	164 (157–173)	0.147	161 (157–169)	170 (161–173)	0.004
% HR max pred	97 (92–102)	93 (91–96)	0.101	92 (91–95)	96 (94–98)	0.004
METs	9.6 (8.9–10.9)	7.0 (6.6–8.1)	<0.001	7.2 (6.5–8.4)	8.9 (7.8–9.9)	<0.001
∆[deoxy(Hb+Mb)] (% ischaemia)	51 (44–57)	37 (28–50)	0.010	36 (28–43)	54 (47–72)	0.005
RPE (6–20)	16 (15–17)	17 (16–18)	0.052	17 (15–18)	17 (17–18)	0.237
Ventilatory thresholds						
${\dot{V}}_{O_{2}\mathrm{GET}}$ (L min^−1^)	1.635 (1.275–1.742)	1.148 (1.005–1.355)	0.001	1.121 (0.975–1.345)	1.423 (1.293–1.675)	<0.001
${\dot{V}}_{O_{2}\mathrm{RCP}}$ (L min^−1^)	1.995 (1.646–2.165)	1.479 (1.283–1.681)	0.002	1.405 (1.286–1.643)	1.767 (1.509–2.129)	<0.001

Median and interquartile range at the peak work rate of the incremental test on a cycle ergometer of the following variables: ${\dot{V}}_{O_{2}}$, pulmonary O_2_ uptake; ${\dot{V}}_{CO_{2}}$, CO_2_ output; RER, gas exchange ratio; ${\dot{V}}_{E}$, pulmonary ventilation; *V*_T_, tidal volume; *f*_R_, breathing frequency; $P_{\mathrm{ET}O_{2}}$, end‐tidal O_2_ partial pressure; $P_{\mathrm{ETC}O_{2}}$, end‐tidal CO_2_ partial pressure; HR, heart rate; METs, metabolic energy of task; ∆[deoxy(Hb+Mb)], deoxygenated Hb+Mb concentrations in vastus lateralis muscle; RPE, rate of perceived exertion. Mean ± SD values of ${\dot{V}}_{O_{2}}$ at the gas exchange threshold (GET) and at the respiratory compensation point (RCP) are also shown. See text for further details.

HR_peak_ corresponded to 96% of the age‐predicted maximum (Tanaka et al., 2001) in Bike Commuters and in Car→Bike Commuters, and to 93% of the age‐predicted maximum in Car Commuters

### 3.6 Measurements in the field: energy expenditure and metabolic CO_2_ output

Data related to the commuting trips, by bicycle or by car, obtained by GPS are presented in Table 4. One‐way trip mean distance was ∼4 km by bicycle and ∼5.5 km by car (*P *= 0.25); mean duration of the trip was ∼16.5 min by bicycle and ∼11 min by car (*P *< 0.001); mean velocity during the trip was ∼15 km h^−1^ by bicycle and ∼28.3 km h^−1^ by car (*P *< 0.001). Mean ${\dot{V}}_{O_{2}}$ determined by the portable metabolimeter was ∼1.5 L min^−1^ during the bicycle commute. This value was slightly lower than the ${\dot{V}}_{O_{2}}$ at GET and corresponded to ∼63% of ${\dot{V}}_{O_{2}\mathrm{peak}}$ in Bike Commuters and to ∼73% of ${\dot{V}}_{O_{2}\mathrm{peak}}$ in Car→Bike Commuters. The ${\dot{V}}_{O_{2}}$ during the car commute was ∼0.4 L min^−1^. Average HR was ∼130 beats min^−1^ during the bicycle commute and ∼85 beats min^−1^ during the car commute. The estimated (by indirect calorimetry) daily and weekly energy expenditures related to the commutes are shown in Table 4. As expected, values were greater in Bike Commuters versus Car Commuters (*P *< 0.001) and in Car→Bike Commuters versus Car Commuters (*P *= 0.001). In Bike Commuters and in Car→Bike Commuters the daily energy expenditure associated with commuting was ∼230 kcal day^−1^, and the weekly energy expenditure was ∼850 kcal week^−1^; this value is slightly lower than the ACSM minimal recommendations for a healthy lifestyle (1000 kcal week^−1^) (Garber et al., 2011).

**TABLE 4:** Measurements in the field: Energy expenditure and metabolic CO_2_ output.

	Bike commuters (*n* = 26)	Car commuters (*n* = 31)	*P*	Car commuters (*n* = 13)	Car→Bike commuters (*n* = 13)	*P*
Weekly bouts (bouts week^−1^)	3 (3–5)	5(5–5)	<0.001	5 (5–5)	2 (2–3)	0.28
Weekly distance (km week^−1^)	25.8 (18.3–36.0)	38.4 (25.5–59.5)	0.015	34.8 (25.7–47.2)	15.0 (10.3–30.7)	0.001
Weekly duration (min week^−1^)	124 (91–143)	88 (72–132)	0.086	84 (75–106)	55 (47–96)	0.048
Single trip distance (km)	3.9 (2.8–4.9)	4.6 (2.8–6.7)	0.252	3.5 (2.6–4.7)	3.2 (2.7–5.7)	0.95
Single trip duration (min)	16 (12–21)	10 (8–14)	<0.001	8 (8–11)	12 (10–19)	0.002
Speed by GPS (km h^−1^)	15.2 (13.2–16.4)	26.8 (21.5–30.5)	<0.001	22.1 (19.8–29.6)	15.5 (13.5–16.6)	0.001
Mean ${\dot{V}}_{O_{2}}$ (L min^−1^)	1.524 (1.372–1.711)	0.446 (0.372–0.493)	<0.001	0.429 (0.348–0.495)	1.512 (1.325–1.744)	0.001
Mean ${\dot{V}}_{CO_{2}}$ (L min^−1^)	1.324 (1.046–1.529)	0.376 (0.313–0.43)	<0.001	0.393 (0.335–0.463)	1.403 (1.256–1.635)	<0.001
Mean ${\dot{V}}_{O_{2}}$ (mL kg^−1^ min^−1^)	22.84 (18.44–25.53)	6.74 (6.02–7.71)	<0.001	6.66 (6.54–7.53)	24.11 (22.06–26.23)	<0.001
Mean HR (b min^−1^)	122 (114–146)	84 (79–91)	<0.001	85 (82–88)	134 (129–149)	<0.001
Mean METs	6.4 (5.3–7.3)	1.9 (1.7–2.2)	<0.001	1.9 (1.9–2.2)	6.9 (6.3–7.5)	<0.001
Mean RER	0.9 (0.9–0.9)	0.8 (0.8–0.9)	0.001	0.8 (0.8–0.9)	0.9 (0.9–0.9)	0.13
RPE	11 (10–12)	6 (6–7)	<0.001	6 (6–7)	12.5 (11.8–13.3)	<0.001
Single trip energy expenditure (kcal)	114 (83–149)	20 (17–31)	<0.001	17 (15–21)	86 (68–138)	0.001
Daily energy expenditure (kcal day^−1^)	227 (167–298)	39 (33–62)	<0.001	34 (30–41)	171 (136–277)	0.001
Weekly energy expenditure (kcal week^−1^)	852 (578–1091)	174 (147–260)	<0.001	170 (149–207)	397 (304–835)	0.001
CO_2_ emission (g km^−1^)	10.1 (8.2–11.7)	1.5 (1.2–2.2)	<0.001	1.9 (1.5–2.5)	9.2 (7.9–11.9)	<0.001

Median and interquartile range data of variables obtained during the commute trips by GPS and by the portable metabolimeter in the different groups of participants. Data of weekly bouts (number of trips, length, duration, distance) of groups bike commuters and car commuters were obtained from questionnaires, whereas in car → bike commuters the data were obtained by the polar flow app during the 24 weeks of intervention. See text for further details.

CO_2_ emission into the atmosphere during the commuting trips was estimated (in g km^−1^) based on the ${\dot{V}}_{CO_{2}}$ measured by the portable metabolimeter (see Methods) (Minetti & Pavei, 2011). The commuting trip by bicycle was performed at a metabolic intensity (freely chosen by the participants) slightly below the gas exchange threshold, that is in the moderate‐exercise intensity domain. Thus, the estimated CO_2_ emission represents only the CO_2_ produced by oxidative metabolism in mitochondria, in the absence of CO_2_ deriving from acid buffering by bicarbonate or from blood CO_2_ stores eliminated by the lungs to compensate for a metabolic acidosis. CO_2_ emission associated with bicycle commuting was 10.1 g km^−1^. We then estimated the total amount of CO_2_ emission ‘saved’ by the participants of the Car→Bike Commuters group, who switched from car to bicycle commuting. Over 24 weeks the participants covered a total of 10,574 km. Considering a petrol car with a standard CO_2_ emission of 108 g km^−1^ (Mellios et al., 2011), the switching from car to bicycle commuting ‘saved’ 1.042 tons of CO_2_ which were not emitted into the atmosphere.

### 3.7 Venous blood samples

Blood biomarkers related to cardiovascular risk, glycaemic control, inflammation, coagulation activity and endothelial function were in the normal range in all groups (Table 5). No significant differences were observed in Bike Commuters versus Car Commuters, except for HDL‐cholesterol, which in the male participants was higher in the Bike Commuters (*P *= 0.02). In the longitudinal study, commuting by bicycle was associated with lower levels of the inflammatory marker fibrinogen (*P *= 0.03), of the glycocalyx component syndecan‐1 (*P *= 0.01) and (in male participants) of the biomarker of myocardial damage troponin I (*P *= 0.04).

**TABLE 5:** Venous blood samples.

	Normal values		Bike commuters (15 F; 11 M)	Car commuters (19 F; 12 M)	*P*	Car commuters (13 F; 12 M)	Car→Bike commuters (13 F; 12 M)	*P*
Erythrocyte sedimentation rate (mm h^−1^)	2–15	F	10.0 (6.1–18.0)	7.0 (5.5–9.5)	0.11	6.1 (5.5–8.4)	7.1 (5.2–9.1)	0.31
	1–10	M	4.5 (4.3–5.2)	5.3 (3.0–0.5)	0.71	5.0 (3.0–6.5)	6.0 (5.5–6.5)	0.40
Interleukin‐6 (pg mL^−1^)	<7	tot	2.1 (2.0–2.1)	2.0 (2.1–2.5)	0.29	2.0 (2.1–2.1)	2.0 (2.1–2.1)	0.26
Pro‐adrenomedullin (nMol L^−1^)	<0.56	tot	0.48 (0.43–0.54)	0.50 (0.46–0.54)	0.65	0.53 (0.48–0.54)	0.5 (0.45–0.6)	0.43
Fibrinogen (nmol L^−1^)	180–380	tot	287 (256–301)	280 (253–335)	0.62	271 (252–344)	238 (226–315)	0.03
HbA1c (mmol mol^−1^)	<48	tot	36.3 (34.3–37.5)	35.6 (34.3–37.5)	0.63	35 (34–37)	36.7 (34.8–38.8)	0.91
DCCT (%)	<6.5	tot	5.4 (5.3–5.6)	5.4 (5.3–5.6)	0.50	5.4 (5.3–5.6)	5.5 (5.3–5.6)	0.82
Creatinin (mg dL^−1^)	0.51–0.95	F	0.75 (0.71–0.78)	0.81 (0.7–0.85)	0.14	0.78 (0.69–0.82)	0.78 (0.73–0.85)	0.07
	0.67–1.17	M	0.98 (0.93–1.05)	0.99 (0.13–0.59)	0.70	1.02 (0.97–1.1)	1.03 (1.01–1.06)	0.90
Total cholesterol (mg dL^−1^)	<200 mg dL^−1^	F	200 (180–215)	176 (167–201)	0.13	194 (176–220)	187 (179–206)	0.55
	<180 mg dL^−1^	M	181 (158–200)	196 (39–0)	0.35	188 (165–228)	189 (176–225)	0.93
HDL cholesterol (mg dL^−1^)	>45 mg dL^−1^	F	65 (59–83)	69 (65–75)	0.44	69 (67–75)	68 (60–77)	0.38
	>35 mg dL^−1^	M	66 (57–73)	54 (9–0)	0.02	57 (47–58)	54 (52–63)	0.34
LDL cholesterol (mg dL^−1^)	<130 mg dL^−2^	F	118 (95–140)	98 (84–118)	0.14	102 (90–139)	101 (86–122)	0.64
		M	98 (78–106)	120 (35–0)	0.16	120 (95–142)	101 (88–148)	0.57
Total cholesterol/HDL cholesterol	<5	tot	2.7 (2.4–3.4)	3 (2–4)	0.32	2.8 (2.5–3.7)	2.8 (2.5–3.4)	0.72
Triglycerides (mg dL^−1^)	40–150	tot	70 (56–90)	70 (59–99)	0.95	70 (60–95)	72 (64–111)	0.22
Homocysteine (µmol L^−1^)	6.0–11.0	F	11.1 (9.3–11.6)	10.6 (9.2–11.6)	0.95	10.6 (9.4–11.7)	9.5 (7.7–11.5)	0.31
	7.4–12.0	M	13.1 (11.2–13.6)	11.3 (3.5–0.3)	0.42	12.6 (11.4–13.8)	13.5 (10.7–17)	0.14
Lipoprotein (a) (mg dL^−1^)	<30	tot	11.9 (4.7–19.1)	7.8 (2–32)	0.44	7.6 (2–29)	13.1 (4.5–32)	0.12
Troponin I hs (ng mL^−1^)	<53.7	F	4.1 (3.1–4.6)	4.6 (3.9–5.3)	0.12	4.9 (4–5.3)	3.6 (3.1–4.7)	0.33
	<78.5	M	7.1 (5.2–7.9)	8.9 (9.3–0.5)	0.64	7.2 (5.1–11.5)	5.7 (4.3–8.9)	0.04
Syndecan‐1 (ng mL^−1^)		tot	16.9 (14.1–21)	21.4 (16.3–29.8)	0.32	21.4 (16.7–29.8)	17.3 (13.8–22.7)	0.01

Median and interquartile range data of blood parameters associated with cardiovascular risk (total cholesterol, LDL‐cholesterol, HDL‐cholesterol, triglycerides, lipoprotein(a), homocysteine, troponin I), glycaemic control (haemoglobin HbA1c, DCCT), coagulation state (fibrinogen), inflammation (interleukin‐6), endothelial function (pro‐adrenomedullin), glycocalyx status (syndecan‐1) in the different groups of participants. See text for further details.

## 4 DISCUSSION

The present study aimed to investigate in healthy adults of both sexes some physiological responses associated with bicycle commuting, over a relatively short distance, compared with car commuting. The aim was to identify beneficial effects of bicycle commuting. More specifically, we hypothesized increased peak aerobic power, exercise tolerance and cardiometabolic fitness, improved microvascular/endothelial function, and skeletal muscle oxidative metabolism. We also hypothesized environmental benefits deriving from bicycle commuting, and specifically significantly reduced CO_2_ emissions in the atmosphere. By identifying these beneficial effects, the ultimate aim of the study was to emphasize the need for educational and urban planning policies aimed at favouring cycling mobility in urban areas.

All the hypotheses mentioned before were confirmed by the obtained results. In short, bicycle commuting had a positive effect on exercise tolerance, cardiorespiratory fitness, microvascular/endothelial function and skeletal muscle oxidative metabolism. Interestingly, these favourable effects were observed also in the longitudinal arm of the study, in which improvements were observed in the participants who switched from car to bicycle commuting.

The study was carried out in a mid‐size town in a flat region of northern Italy. An ‘active’ group of subjects (Bike Commuters) was tested. These participants were commuting by bicycle, covering a mean one‐way home–work distance of ∼4 km in ∼17 min, 4–5 times per week, at an average self‐selected speed of ∼15 km h^−1^, corresponding to a ${\dot{V}}_{O_{2}}$ of ∼1.5 L min^−1^ (∼60 % of ${\dot{V}}_{O_{2}\mathrm{peak}}$), slightly lower than the individuals’ first ventilatory threshold (GET). In other words, the exercise stimulus associated with bicycle commuting was of moderate intensity. Within a cross‐sectional comparison, these active participants were compared with a group of ‘sedentary’ participants (Car Commuters), who did a commute of similar distance by car. In a longitudinal leg of the study, a subgroup of Car Commuters agreed to commute by bicycle for 6 months, and was re‐evaluated after this intervention (Car→Bike Commuters). All participants were free of significant diseases, were not obese or overweight, had normal blood pressure and at routine blood examination did not show evidence of increased cardiovascular or metabolic risk. Physically very active individuals and athletes were excluded from the study. The habitual level of physical activity (IPAQ scores) was in the ‘minimally active’ or ‘inactive’ categories. Apart from the physical activity associated with bicycle commuting, the IPAQ scores of Bike Commuters and Car Commuters were not different.

Bike Commuters had a better body composition compared to Car Commuters: for the same BMI, the %FFM was higher and the %FM was lower in Bike Commuters. On the other hand, the %FFM and the %FM were not different in Car→Bike Commuters versus Car Commuters. The data suggest that bicycle commuting had a positive effect on body composition, although 6 months was not long enough to induce favourable changes of these variables in the longitudinal evaluation (Car→Bike Commuters vs. Car Commuters). We do not know of previous studies specifically investigating this aspect. The present study did not comprehend dietary interventions, nor a detailed analysis of dietary habits of the participants.

Bike Commuters had a greater exercise tolerance (higher peak work rate and peak aerobic power (${\dot{V}}_{O_{2}\mathrm{peak}}$), higher ventilatory thresholds (the fractions of ${\dot{V}}_{O_{2}\mathrm{peak}}$ which can be sustained by relatively long periods of time) versus Car Commuters, demonstrating greater cardiorespiratory fitness. An increased peak fractional O_2_ extraction by skeletal muscle (see the Δ[deoxy(Hb+Mb)]_peak_ data determined by NIRS) contributed to higher performance of Bike Commuters. Whereas cardiac output and peak cardiovascular function were not determined in the study, microvascular/endothelial function was specifically investigated (see the PLM test), and it was found to be improved in Bike Commuters versus Car Commuters. This observation is of significant interest, considering the role played by microvascular/endothelial impairments in the development of cardiovascular diseases, insulin resistance and other chronic diseases (Versari et al., 2009; Walker et al., 2019). The hyperaemic response determined by the PLM test is known to be significantly impaired in old subjects (Groot et al., 2016), in patients with cardiac (Witman et al., 2015) or pulmonary (Iepsen et al., 2016) or other diseases (Gifford & Richardson, 2017). The present study demonstrates that the microvascular/endothelial function evaluated by the PLM test seems quite respondent to a training stimulus in healthy subjects. Going in the other direction (detraining), we recently demonstrated, by the PLM test, an acute impairment of microvascular/endothelial function in young healthy subjects undergoing a short period of disuse/microgravity (bed rest) (Zuccarelli et al., 2021).

Positive effects associated with bicycle commuting were also noticed for the blood biochemistry data. Specifically, a significant reduction of syndecan‐1 was observed in Car→Bike Commuters versus Car Commuters, paralleled by a significant reduction of the inflammatory biomarker fibrinogen. It is known that a sedentary lifestyle is associated with a low‐grade systemic inflammation and with the elevation of the acute‐phase protein fibrinogen, whose levels are associated with mortality (Fibrinogen Studies Collaboration et al., 2005). Moreover, an erosion of the glycocalyx, assessed as an increase in syndecan‐1 circulating levels (Dognè & Flamion, 2020), occurs in an inflammatory milieu, and is coupled with alterations of vascular permeability, reduced shear stress‐induced release of nitric oxide, vascular inflammation and increased risk of thrombi (Kei et al., 2023). The data of the present study are supported by those deriving from large cohort studies, showing that physical activity reduces fibrinogen levels (Gomez‐Marcos et al., 2014). Lastly, in the male participants of the present study we observed a significant reduction of troponin I levels in Car→Bike Commuters versus Car Commuters. This observation is of interest, since troponin I levels, assessed by high sensitivity assays, are associated with cardiovascular mortality in the general population, even if they fall below the 99th percentile of the reference population (Aakre & Omland, 2019).

Taken together, the observations mentioned above fit with a concept put forward in a previous epidemiological longitudinal study, indicating that bicycle commuting exerts positive effects on life expectancy and development of cardiovascular, neoplastic, mental and other diseases (Friel et al., 2024).

As mentioned above, the results of the present study are novel and should be of interest also because the training stimulus associated with bicycle commuting was moderate. The distance covered was indeed relatively short (∼4.5 km, ∼17 min one‐way), and was carried out at moderate intensity (slightly below GET). Nonetheless, the daily (∼250 kcal) and weekly (∼850 kcal) energy expenditures associated with the commute trips by bicycle were close to the ACSM recommendations for the minimum physical activity level associated with a healthy lifestyle (1000 kcal week^−1^) (Garber et al., 2011).

The estimated CO_2_ emission in the atmosphere during bicycle commuting was ∼10 g km^−1^, that is ∼10 times less compared to the emission associated with a hybrid car, and ∼12 times less than that associated with a petrol car (Minetti & Pavei, 2011). This demonstrates a very substantial decrease of CO_2_ emission in the atmosphere associated with bicycle commuting versus commuting by car, also after considering that 4–5 people could fit in a single car. The CO_2_ emission during bicycle commuting was also lower than that determined by Minetti and Pavei (2011) for running subjects, and slightly lower than that determined by the same authors on walking subjects. This is no surprise, since it is well known that the energy cost of locomotion (the metabolic output per unit of distance) is substantially lower in cycling compared to running or walking (di Prampero, 1986).

### 4.1 Limitations

The study has some limitations, which should be recognized also in order to envisage future studies to be performed.

In the present study we utilized the term ${\dot{V}}_{O_{2}\mathrm{peak}}$, that is the ${\dot{V}}_{O_{2}}$ reached at voluntary exhaustion during an incremental test. ‘${\dot{V}}_{O_{2}\max}$’ could not be used because a plateau in ${\dot{V}}_{O_{2}}$ was not observed at the highest loads. A ‘verification’ exercise (Poole & Jones, 2017) was not performed. The verification phase consists of a square‐wave bout of slightly supramaximal exercise performed a few minutes after the termination of the incremental test. The aim is to check the presence of increases of ${\dot{V}}_{O_{2}}$ values compared to ${\dot{V}}_{O_{2}\mathrm{peak}}$. According to the meta‐analysis by Costa et al. (2021), however, in healthy subjects the ${\dot{V}}_{O_{2}\mathrm{peak}}$ increase detectable by the verification phase is on average less than 1%, which is substantially lower than the test variability for these measurements. Moreover, in the present study the differences observed between groups for ${\dot{V}}_{O_{2}\mathrm{peak}}$ were confirmed also when the ventilatory thresholds (GET and RCP) were considered. GET and RCP determination do not rely on the subject performing a maximal effort.

Although the power analysis confirms the presence of an adequate number of participants, a larger sample size, with a wider age distribution, would be of interest, by allowing the broadening of the inferences of the results on ageing subjects. The relatively limited number of subjects did not allow us to evaluate differences in responses between males and females. A longer follow‐up during the longitudinal arm of the study would be of interest as well, possibly allowing detection of a minimum duration of commuting by bicycle capable of exerting effects on the variables. Recruitment of participants with some pathological conditions could expand the inferences of the study in terms of public health. Of particular interest would be patients with initial signs/symptoms of cardiovascular or metabolic diseases. As mentioned above, a formal analysis of the dietary habits of the participants was not performed. In association with bicycle commuting, dietary interventions could have a positive synergic effect on the investigated variables. Dietary habits could also have relatively minor effects on ${\dot{V}}_{CO_{2}}$ and RER measurements. The potential negative consequences of an increased pulmonary ventilation on the inhalation of airborne pollutants have not been evaluated. Other variables (cardiovascular responses, mitochondrial respiration) could be determined, allowing a more comprehensive evaluation of the integrated responses of the cardiorespiratory and muscular systems. Lastly, the data obtained in the present study are representative of a specific sample of people living in an urban context in a northern Italian area with medium–low population density, flat terrain and infrastructure that supports bicycle use. Replicating the study in different urban contexts would be of interest, also in terms of inferences on public health.

### 4.2 Conclusions

We investigated in healthy adults of both sexes the physiological responses associated with bicycle commuting over a relatively short distance (∼4.5 km, ∼17 min one‐way, 4–5 times per week), compared with car commuting. Both a cross‐sectional and a longitudinal experimental approach were utilized. Although the training stimulus associated with bicycle commuting was moderate, we demonstrated clear beneficial effects of bicycle commuting in increasing maximal aerobic power and exercise tolerance, improving microvascular/endothelial function, skeletal muscle oxidative metabolism, cardiometabolic fitness and body composition. Blood biochemistry variables provided evidence of a reduced cardiovascular risk. CO_2_ emission into the atmosphere was substantially reduced (10–12 times lower) during bicycle commuting vs. commuting by car. The obtained results stress the need for educational and urban planning policies aimed at favouring cycling mobility in urban areas.

## AUTHOR CONTRIBUTIONS

B.G. and F.F. conceived the study and obtained the funding for the PhD scholarship to C.U. C.U. was responsible for the coordination and the conduction of the experiments. C.U., G.B., L.Z., F.C., F.C., M.dA., M.d.M., E.F., A.P. and A.P.B. participated in the experiments, data analysis and interpretation. C.U. and B.G. wrote the first draft of the manuscript. All authors participated to the revision of the initial draft. All authors have read and approved the final version of this manuscript and agree to be accountable for all aspects of the work in ensuring that questions related to the accuracy or integrity of any part of the work are appropriately investigated and resolved. All persons designated as authors qualify for authorship, and all those who qualify for authorship are listed.

## CONFLICT OF INTEREST

None declared.

## Data Availability

The datasets used and analysed during the current study is available from the corresponding author on reasonable request.
